# Use of strain and shear wave elastography in the ultrasonographic evaluation of the intermediate patellar ligament in horses: comparison and consistency of data

**DOI:** 10.3389/fvets.2026.1817578

**Published:** 2026-05-20

**Authors:** Paola Straticò, Lorenza Bandera, Giulia Guerri, Andrea Agosta, Irene Paolacci, Lucio Petrizzi, Massimo Vignoli, Vincenzo Varasano

**Affiliations:** Department of Veterinary Medicine, University of Teramo, Teramo, Italy

**Keywords:** horse, intermediate patellar ligament, shear wave elastography, stifle, strain elastography, ultrasonography

## Abstract

The clinical significance and ultrasonographic features of intermediate patellar ligament (IPL) desmopathy in horses are still debated and considered nonspecific. Elastosonography allows assessment of tissue elasticity in response to an external stimulus. The aim of the study was to describe the elastosonographic characteristics of the equine IPL, evaluate the feasibility of both strain (SE) and two-dimensional shear wave elastography (2D-SWE), and assess the consistency of the data obtained from these techniques. 20 adult horses of mixed breed, sex and athletic use were selected and allocated to a Sound Group (GS, n.15) or a Lame Group (GL, n.5) based on orthopedic examination findings, according to the presence a stifle-originating lameness and/or with ultrasonographic evidence of IPL lesion. SE and 2D-SWE of both IPLs were performed in longitudinal and transverse scans. Using the same Region of Interest (ROI), SE evaluated the Elasticity Index of the IPL (EI_IPL_) and the Strain Ratio (SR) between the IPL and infrapatellar fat pad, while 2D-SWE measured Shear Wave Velocity (m/s) and Young’s modulus (kPa). Feasibility of both techniques was good, with acceptable to good Intraclass Correlation Coefficient (0.7 < ICC < 0.9) (*p* < 0.05). No significant differences were found between left and right limb, or between intraoperator measurements (Wilcoxon test). No correlation was observed between SE variables (EI and SR) or SWE variables (m/s and kPa) and IPL diameter/thickness ratio (Pearson correlation). SE was able to differentiate between GS and GL, with an EI of the IPL that was statistically higher in GS in transverse scan (respectively, 1.9 and 1.7; *p* = 0.001). Equine IPL can be efficiently investigated using SE and 2D-SWE. It exhibits elastosonographic characteristic of a hard, non-deformable structure, with increased stiffness in cases of chronic injuries.

## Introduction

Stifle disorders in sport horses account for 8–42% of hindlimb lameness, with different etiologies depending on the horse’s athletic use, conformation, age and level of work (1–4).

Patellar ligament desmopathy have been described in 4–18% of horses undergoing stifle ultrasonography (5–8), likely due to improved access to advanced imaging equipment rather than increased prevalence of the disorder (9). Technological improvement has also enabled the identification of breed-related ultrasonographic differences in the intermediate patellar ligaments between Quarter Horses and Warmbloods (e.g., striation pattern and width, ligament cross sectional area) (10).

Despite the ultrasonographic evidence of patellar ligaments lesions, their clinical significance is still debated (8). Clinical signs associated with patellar ligament desmopathy are nonspecific and mainly related to mild chronic lameness and poor performance, peripatellar edema or thickening of the ligament itself (5). Unlike lesions of the lateral patellar ligament which are mainly traumatic, those affecting the intermediate patellar ligament appear to have a degenerative pathophysiology and are more commonly located at its medio-distal aspect (1, 7, 11).

Due to the topographic anatomy of the three patellar ligaments in horses, the intermediate (IPL) shares many similarities with the patellar ligament in humans (12), with normal homogeneously hyperechoic ultrasonographic appearance and an oval to triangular shape in transverse scan (5).

Tissue elasticity can be quantified in response to external stimuli (13). Elastography allows differentiation between normal and diseased tissues based on differences in their elastic properties (14, 15). Strain Elastography (SE) uses manual compression applied by an operator through the probe, and displays tissue elastic properties as an elastogram, quantified using the elasticity index (EI) and the Strain Ratio (SR) between the target tissue and a reference tissue (16). In two-dimensional Shear Wave Elastography (2D-SWE), shear waves are automatically generated and their propagation velocity within the tissue is analyzed according to its viscoelastic properties (17, 18), finally generating an elastogram, which is a colored representation of tissue response to the impulse (19). So far, while SE has gained popularity as an aid to investigate tendon and ligaments (20–26) both in human and veterinary orthopedics. 2D-SWE musculo-skeletal application remains limited, particularly in veterinary medicine (27–29), although it has been used to investigate breast and thyroid lesions and liver fibrosis (30–33).

Aim of this study was to describe the ultrasonographic characteristics of the IPL using SE and SWE in horses and to assess the feasibility of the techniques and their accuracy in the identification of horses affected and not affected by IPL desmopathy.

## Materials and methods

All procedures were approved by the Local Ethical Committee (Protocol n. 11/2019). Privately-owned horses referred to the Veterinary Teaching Hospital were prospectively recruited, and an informed consent was obtained by the owner. Adult horses of mixed sex, breed and athletic use undergoing a whole clinical and orthopedic examination, radiographic and B-mode ultrasonographic screening were included in the study. Those who were free from lameness and had an unremarkable radiographic and ultrasonographic exam of the stifle were allocated to the Group Sound (GS). Horses were assigned to the Lame Group (GL) if they exhibited hindlimb lameness that was specifically localized to the stifle region based on a comprehensive diagnostic work-up. This included a complete clinical and orthopedic examination, followed by intra-articular anesthesia of the stifle that resulted in a clear improvement or resolution of lameness. Only horses showing radiographic and ultrasonographic findings consistent with IPL disorder, without evidence of other clinically relevant stifle abnormalities, were included in this group. Age, breed, gender and athletic use were recorded in the medical report of each horse. For diagnostic imaging purposes, horses were sedated with xylazine (0.5 mg/kg i.v.) and examined in a standing position with both hindlimbs in a full weight-bearing stance. Radiographic examination included lateromedial and caudocranial projections of each stifle (M.T. Medical Technology CS01MS). For the ultrasonographic exams, a high frequency linear probe (8.5–10 MHz) connected to an ultrasound system (Logiq S8XD Clear. GE) was used. A complete B-mode evaluation of the femorotibial and femoropatellar joints, including menisci, collateral ligaments, and patellar ligaments, was performed.

SE and SWE were conducted on the midbody of the IPL using both longitudinal (parallel to fiber orientation) and transverse (perpendicular to fiber orientation), with the limb maintained in weight-bearing conditions, by two experienced operators, named OP1 and OP2 (G.G. and P.S.). The IPL diameter (transverse scan) and cranio-caudal thickness (longitudinal scan) were recorded, and their ratio calculated. The standoff pad was not used during any procedure.

**Figure 1:**
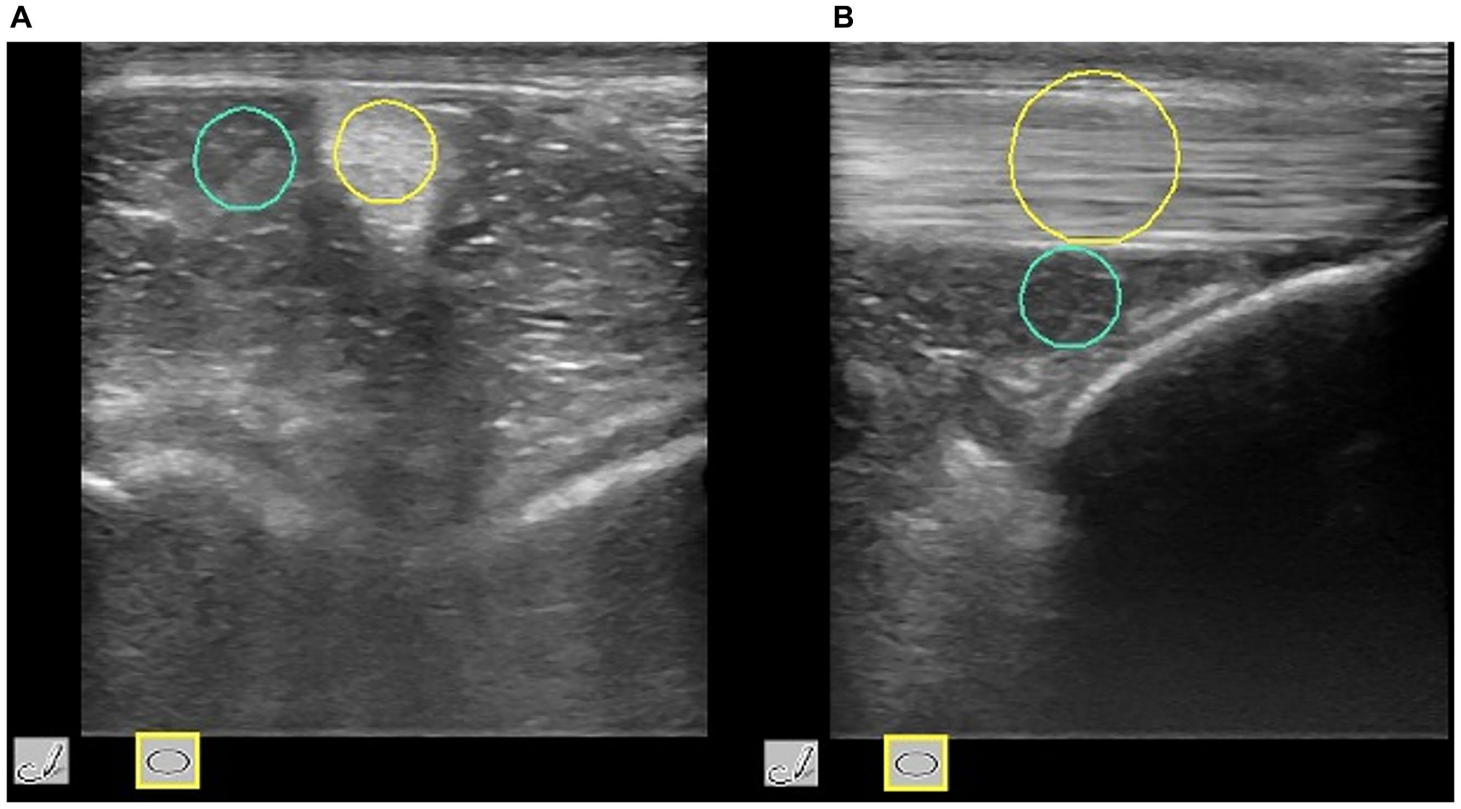
Transverse **(A)** and longitudinal **(B)** scans of the IPL. For SE in transverse scan the ROI of the IP (yellow circle) was positioned over the largest area of the IPL, and had a diameter equal to its largest diameter; in longitudinal scan the ROI was positioned over the midbody of the IPL and had a diameter equal to its cranio-caudal thickness (SE: Strain Elastography; IPL: Intermediate Patellar ligament; ROI: Region of Interest; green circle: ROI placed over the peripatellar fat pad).

**Figure 2:**
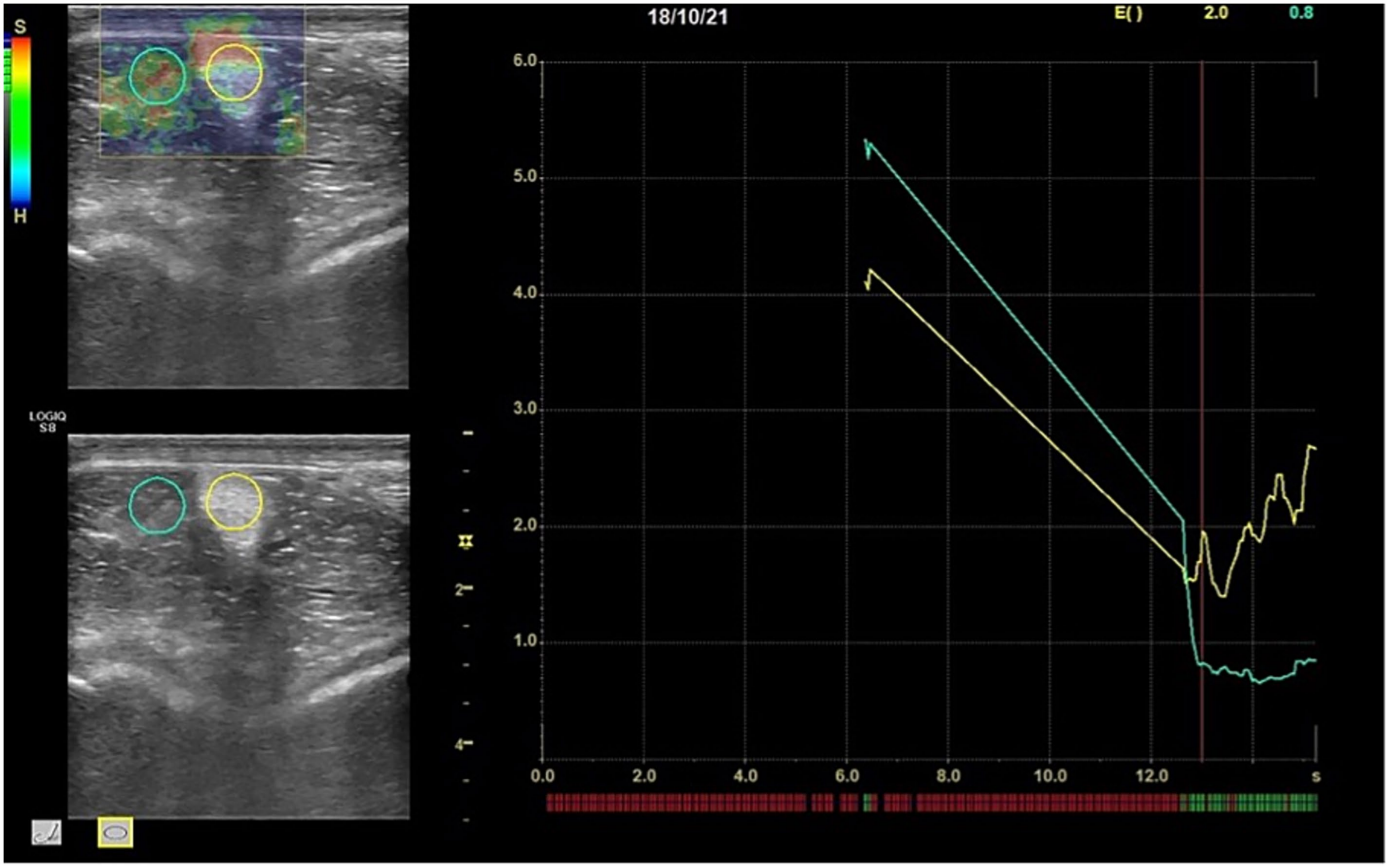
Software output showing the EI of the IPL (yellow circle) and the infrapatellar fat pad (green circle) in transverse scan. Once the EI of both structures are calculated, the software automatically gives the value of the SR. At the top right elasticity index (E) and strain ratio values are shown in yellow and green, respectively. At the bottom of the right diagram red lines stand for low quality of the image unsatisfactory for the measurement, whereas the green lines indicate an image suitable for a measurement. The yellow line refers to the IPL strain, the green line to the infrapatellar fat pad. The x axis shows time in seconds and the y axes the elasticity index of each structure (EI: Elasticity Index; IPL: Intermediate Patellar Ligament; ST: Strain Ratio).

For SE, each operator executed rhythmic and regular low-frequency compression and relaxation cycles with the probe over the area of interest. To obtain an appropriate image, the exerted pressure was moderate (i.e., level of pressure sufficient to maintain skin contact) and adjusted according to the strain-bar indicator on the lateral part of the elastogram. Moreover, to minimize inter-operator variations, each SE scan was repeated until at least three compression-decompression cycles of optimal strain were obtained at the region of interest (ROI). Scans were assumed suitable when the strain-bar indicator consistently displayed a green color. Measurements were taken three times per scan. Elastosonographic images of both OPs were analyzed by one observer blinded to the group to which the horses were assigned. To compare SE measures with SWE measures, only a quantitative method was used to evaluate the elastograms (23). A circular ROI was placed over the IPL midbody, with size adapted to the ligament dimensions (i.e., equal to IPL diameter in transverse scans and to cranio-caudal thickness in longitudinal scans), thus not fixed across all acquisitions but standardized relative to ligament size (Figure 1). The Elasticity Index of the Intermediate Patellar Ligament (EI_IPL_) over the selected ROI and the Strain Ratio (SR) between the IPL and a reference tissue (the infrapatellar fat pad) were calculated (Figure 2).

**Figure 3:**
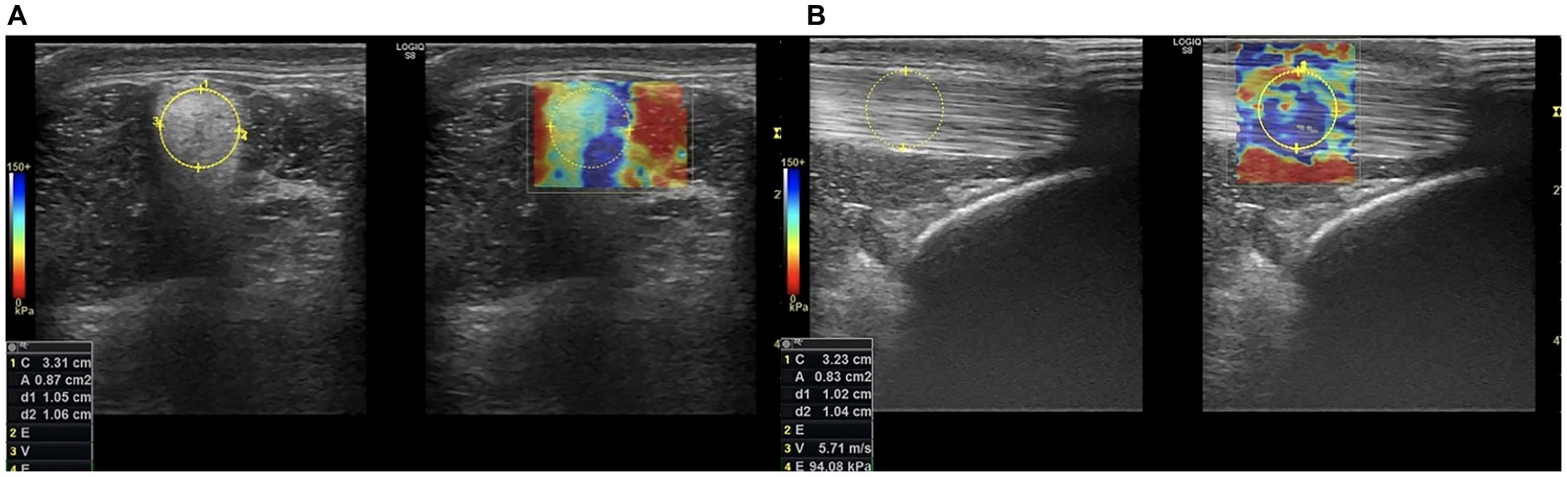
Transverse **(A)** and longitudinal **(B)** scans of the IPL. For SWE transverse scan the ROI of the IPL was positioned over the largest area of the IPL and had a diameter equal to its largest diameter; in longitudinal scan the ROI was positioned over the midbody of the IPL and had a diameter equal to its cranio-caudal thickness. After placing the ROI, the software calculated the velocity (m/s) and the Youngs modulus (kPa), both showed in the box at the left bottom side of the scans (SWE: Shear Wave Elastography; IPL: Intermediate Patellar Ligament).

**Figure 4:**
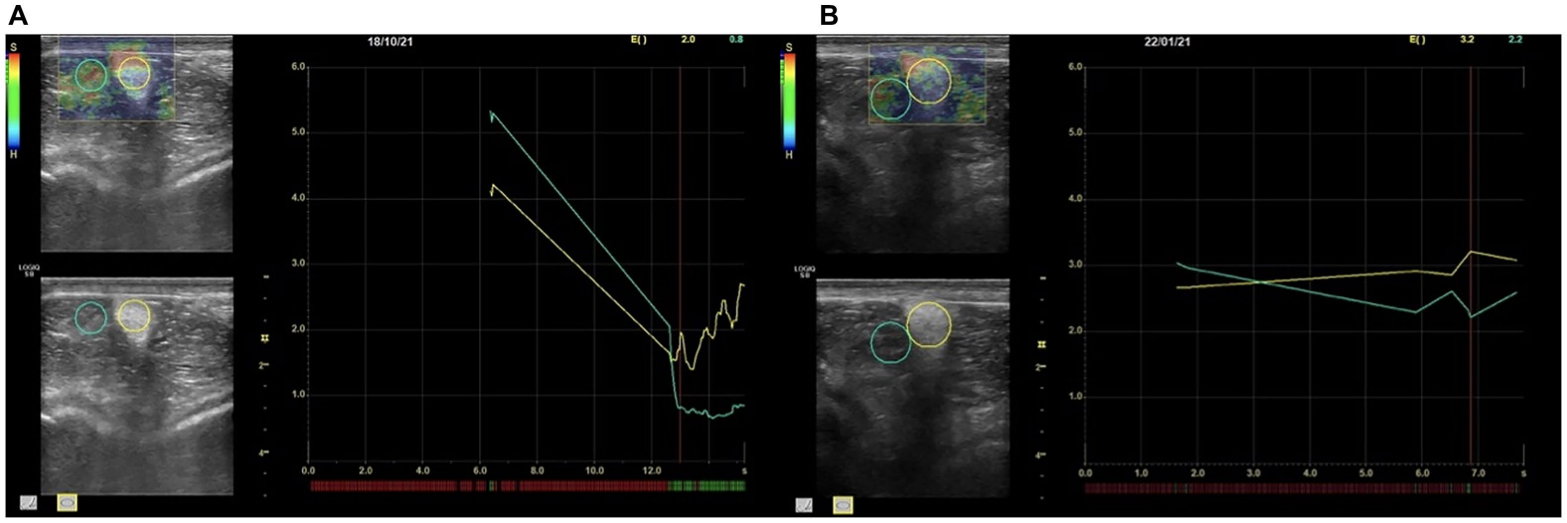
SE transverse scan of the IPL of a horse belonging to GS **(A)** and a horse belonging to GL **(B)**. At the top right elasticity index (E) and strain ratio values are shown in yellow and green, respectively. At the bottom of the right diagram red lines stand for low quality of the image unsatisfactory for the measurement, whereas the green lines indicate an image suitable for a measurement. The yellow line refers to the IPL strain, the green line to the infrapatellar fat pad. The *x* axis shows time in seconds and the *y* axes the elasticity index of each structure (SE: strain elastography; IPL: intermediate patellar ligament; GS: group sound; GL: group lame).

For SWE, the probe was held still, and 5–10 cycles were recorded for each scan. Elastosonographic images were analyzed by one observer blind to the group to which the horses were assigned. The ROI was positioned identically to SE in terms of location and size criteria (Figure 3). The software calculated the Elasticity Index (EI), the velocity (m/s) and the Young’s modulus (kPa) of the IPL for each scan (Figure 4). As for SE, each measurement was repeated 3 times and recorded.

Data were collected on electronic spreadsheets (Excel for Mac, Version 16.94) and analyzed with IBM SPSS Statistics software (IBM SPSS, v.29.0).

Normality of data was assessed with the Shapiro–Wilk test.

Although image acquisition was guided by real-time quality indicators provided by the ultrasound system, a small number of scans were excluded during post-processing because the stored images did not meet the quality criteria required for reliable quantitative analysis. Feasibility of each technique was thus evaluated by assessing the number of not diagnostic exams. Nonparametric statistics was used to compare data from OP1 and OP2 or from the same operator (Mann–Whitney U test and Friedman test), or between left and right limbs (Wilcoxon test). Each measurement was repeated 3 times by two operators and intraoperator agreement was evaluated. Intra-Correlation Coefficients (IntraCC) were calculated based on a mean-rating (k = 3), absolute agreement, 2 way random-effect model.

Correlations between the SE variables (EI and SR) or SWE variables (m/s and kPa) and IPL diameter/thickness ratio were analyzed with a Pearson’s correlation test. Comparisons between left and right limb were included in the analysis (Friedman test).

The ability of SE and SWE to differentiate between healthy and diseased IPLs was assessed by investigating the differences of the variable between GS and GL (Mann–Whitney U test).

In all cases, statistical significance was set at *p* < 0.05.

## Results

Twenty horses were included in the study mixed for sex (8 females, 5 stallions, 7 geldings), breed (9 Quarter Horses, 4 Arabian Thoroughbred, 4 saddle breeds, 1 Paint Horse, 1 Appaloosa, 1 Haflinger) and athletic use, with median age 6 years (range 2–20).

Based on clinical and ultrasonographic examinations, 15/20 (75%) were assigned to the GS group and 5/20 (25%) to the GL group. Groups did not differ statistically for age (GS mean 7.8 years, median 6, range 2–20; GL mean 7.2 years, median 3, range 3–14; Mann–Whitney U test, *p* > 0.05).

Concerning the feasibility of the study, scans were not diagnostic for SWE analysis in 7/80 acquisitions for OP1 (4/40 in longitudinal and 3/40 in transverse scans) and 4/80 for OP2 (2/40 in longitudinal and 2/40 in transverse scans). In case of SE analysis, 6/80 acquisitions for OP1 (3/40 longitudinal and 3/40 transverse scans) and 4/80 for OP2 (2/40 longitudinal and 2/40 transverse scans) were unsuitable to measure EI_IPL_. When the EI _FAT_ and SR were calculated, 6/80 acquisitions (4 longitudinal and 2 transverse scans) for OP1 and 5/80 (3 longitudinal and 2 transverse scans) for OP2 were unsuitable.

Table 1 summarizes IPL diameter (in transverse scans), its cranio-caudal thickness (in longitudinal scans) and the ratio between the two, $\frac{\text{diameter}}{\text{thickness}.}$

**Table 1:** IPL diameter in transverse scan, its cranio-caudal thickness in longitudinal scan and the ratio $\frac{\text{diameter}}{\text{thickness}}$ .

Operator	Limb	Type of measure	Mean ± SD (cm)
OP1	Left	Diameter	0.92 ± 0.22
	Right	Diameter	0.86 ± 0.08
OP1	Left	Thickness	0.81 ± 0.14
	Right	Thickness	0.82 ± 0.12
OP1	Left	Ratio	1.16 ± 0.24
	Right	Ratio	1.07 ± 0.17
OP 2	Left	Diameter	0.91 ± 0.35
	Right	Diameter	0.89 ± 0.07
OP2	Left	Thickness	0.85 ± 0.13
	Right	Thickness	0.8 ± 0.11
OP2	Left	Ratio	1.13 ± 0.16
	Right	Ratio	1.08 ± 0.31

Values are expressed as mean ± SD (OP: operator; IPL: intermediate patellar ligament; SD: standard deviation).

Both SE and SWE measurement showed an intraclass correlation coefficient (intra-CC) from acceptable to good (0.7 < ICC < 0.9) (*p* < 0.05). For SE, the comparison of the 3 measures taken by each operator on the whole sample showed significant differences for the EI_IPL_ and EI_FAT_ in transverse scan of the left limb for OP1 (*p* = 0.04 and *p* = 0.006 respectively). The same evaluation was done on GS and GL, with significant differences in the GS group for the EI_FAT_ of the left limb for OP1 (*p* = 0.007), and of the right limb for OP2 (*p* = 0.001) (Friedman test). For SWE, no intraoperator significant difference was found (*p* > 0.05) (Wilcoxon test), nor for m/s or for kPa, in any scan.

To evaluate between-operator differences, a Mann Whitney U test was used. For SE a significant difference was found between OP1 and OP2 for the SR of the right limb in transverse scan (OP1: median = 3, mean = 3, max = 5, min = 3; OP2: median = 1, mean = 1.78, max = 6.3, min = 0.1; *p* = 0.01).

For SWE, interoperator repeatability was also assessed, without any statistical difference, for m/s and kPa (*p* > 0.05).

A comparison between left and right limb for both techniques was made on the whole sample as well on GS and GL without any relevant difference for SE nor SWE (*p* > 0.05).

When comparing GS and GL, strain elastography (SE) revealed a significant difference exclusively in the Elasticity Index of the IPL (EI_IPL_) in transverse scans. No significant differences were detected for the other SE variables. In contrast, shear wave elastography (SWE) did not show any significant differences between groups for any of the evaluated parameters (*p* > 0.05). Values of EI_IPL_, SR, shear wave velocity (m/s), and Young’s modulus (kPa) for both groups are reported in Table 2. Pearson’s correlation of the variables with the diameter to thickness ratio $\frac{\text{diameter}}{\text{thickness}}$ were weak and mostly not significant. Only 3 significant correlations were observed for SWE in OP1 between the ratio and m/s and kPa values in transverse scan for the left limb and m/s of the right limb in longitudinal scan. Concerning SE, EI_IPL_ in transverse scan of the right limb for OP1 and SR in transverse scan of the left limb for OP2 showed significant but weak correlations with the ratio (Table 3).

**Table 2:** EI_IPL_, SR, m/s, and kPa in GS and GL $\frac{\text{diameter}}{\text{thickness}}$ .

	Scan	GS Median (minimum−maximum)	GL Median (minimum−maximum)	*p*
EI_IPL_	Transverse	1.9 (0.6–5.6)^a^	1.7 (0.6–4.8)^b^	*0.001*
EI_IPL_	Longitudinal	1.8 (0.7–5.5)	2.2 (0.8–5.4)	0.9
SR	Transverse	1.15 (0.1–6.8)	1.5 (0.2–5.4)	0.06
SR	Longitudinal	2.35 (0.1–6.8)	1.5 (0.1–5.3)	0.3
m/s	Transverse	4.9 (1.75–7.39)	4.89 (2.78–6.24)	0.30
m/s	Longitudinal	4.9 (1.53–7.39)	4.89 (2.78–6.24)	0.14
kPa	Transverse	73.61 (23.21–140.6)	71.68 (23.21–140.6)	0.77
kPa	Longitudinal	73.61 (7.26–1240.67)	72.28 (23.21–116.36)	0.34

Values are expressed as median and range minimum-maximum (EI_IPL_: Elasticity Index of the Intermediate Patellar Ligament; SR: Strain Ratio; GS: sound group; GL: lame group). Statistical significance was set at *p* < 0.05. Different letters in the same row indicate significantly different results (*p* < 0.05). The meaning of italic values is diameter/thickness: ratio between the diameter of the IPL in transverse scan, and its craniocaudal thickness in longitudiunal scan.

**Table 3:** Correlations of the variables (m/s and kPa for SWE; EI_IPL_ and SR or SE) with the diameter to thickness ratio $\frac{\text{diameter}}{\text{thickness}}$ in the whole sample in longitudinal and transverse scans (m/s: velocity; kPa: Young’s Modulus; EI_IPL_: Elasticity Index of the Intermediate Patellar Ligament; SR: Strain Ratio; *r*: Pearson’s correlation index).

	*SWE*			*SE*		
Limb and OP		*r*	*p*		*r*	*p*
Left OP1	m/s transv	−0.51	*0.02*	EI_IPL_ transv	0.17	0.49
	m/s long	−0.08	0.75	EI_IPL_ long	−0.08	0.74
	kPa transv	−0.54	*0.02*	SR transv	0.07	0.79
	kPa long	−0.03	0.91	SR long	−0.24	0.34
Right OP1	m/s transv	−0.28	0.24	EI_IPL_ transv	0.51	*0.03*
	m/s long	0.46	*0.04*	EI_IPL_ long	0.29	0.27
	kPa transv	−0.29	0.21	SR transv	−0.13	0.62
	kPa long	−0.41	0.07	SR long	−0.40	0.11
Left OP2	m/s transv	−0.13	0.06	EI_IPL_ transv	0.35	0.13
	m/s long	−0.01	0.96	EI_IPL_ long	0.44	0.06
	kPa transv	−0.14	0.56	SR transv	−0.48	*0.03*
	kPa long	−0.05	0.82	SR long	−0.26	0.28
Right OP2	m/s transv	−0.22	0.33	EI_IPL_ transv	−0.08	0.73
	m/s long	−0.09	0.69	EI_IPL_ long	0.08	0.74
	kPa transv	−0.26	0.25	SR transv	0.03	0.91
	kPa long	−0.08	0.74	SR long	0.02	0.92

Statistical significance was set at *p* < 0.05. The meaning of italic values is diameter/thickness: ratio between the diameter of the IPL in transverse scan, and its craniocaudal thickness in longitudiunal scan.

Table 4 summarizes the descriptive statistics of the measured variables with both techniques in GS and GL.

**Table 4:** Descriptive statistics of the measured variable in GS and GL with SWE and SE $\frac{\text{diameter}}{\text{thickness}}$ .

		GS		GL	
		Left Median (Minimum−Maximum)	Right Median (Minimum−Maximum)	Left Median (Minimum−Maximum)	Right Median (Minimum−Maximum)
SWE	m/s transv	4.76 (2.78–6. 53)	5.03 (1.75–7.39)	5.13 (2.25–6.86)	4.68 (2.78–6.24)
	m/s long	4.94 (1.53–8.35)	5.04 (2.23–7.39)	5.24 (0.7–5.74)	4.66 (2.78–6.24)
	kPa transv	69.76 (15.98–129.02)	76.85 (9.34–165.12)	71.69 (38.86–140.60)	65.67 (23.21–116.36)
	kPa long	71.92 (7.26–180.35)	75.48 (15.12–165.12)	85.4 (28.39–98.34)	66.7 (23.21–116.36)
SE	EI_IPL_ transv	1.9 (0.7–5.1)	1.95 (0.6–5.6)	1.7 (0.8–2.2)	2 (0.6–4.8)
	EI_IPL_ long	1.08 (0.9–5.4)	1.95 (0.7–5.5)	2.2 (0.9–4.6)	2.9 (0.8–5.4)
	SR transv	2.5 (0.2–4)	2.48 (0.2–4)	1.8 (0.5–5.4)	1 (0.2–5.4)
	SR long	2.5 (0.1–6)	2.3 (0.2–6.8)	1.5 (0.3–4.7)	1.4 (0.1–5.3)

Values are expressed as median and range minimum-maximum (GS: Sound group; GL: Lame group; SWE: Shear Wave Elastography; SE: Strain Elastography; m/s: velocity; kPa: Young’s Modulus; EI_IPL_: Elasticity Index of the Intermediate Patellar Ligament; SR: Strain Ratio).

## Discussion

Since intermediate patellar ligament desmopathy occurs more often at the midbody region rather than at the enthesis (7), we aimed to describe its elastosonographic characteristics at this level, using SE and SWE in a cohort of horses unaffected by IPL pathology and a cohort diagnosed with IPL lesions. The infrapatellar fat pad, which surrounds the ligaments and contributes to joint homeostasis, has been implicated as a source of immune cells and inflammatory mediators in osteoarthritis (34, 35). It was appointed as the SE reference tissue because it has shown to be superior to other tissues (i.e., skin) when evaluating Achille’s tendons in humans (36, 37). Even though it can be considered a reliable structure and taken as reference region, its unknown elastic pattern in horses cannot exclude alterations due to individual characteristics or underlying pathologies, as in Hoffa’s pad impingement syndrome (38) in humans, where swelling, hypertrophy of fibrosis can interfere with the measurements.

Consistent with previous work (20, 24, 25, 27, 29, 39, 40) both techniques showed acceptable to good repeatability, with SWE proving more robust than SE when repeated measurements from the same operator or from different operators. Looking in detail at the results, SE showed significant differences for EI of the IPL and fat in transverse scan, likely reflecting the high deformability of the infrapatellar fat pad and its susceptibility to artifacts, which can reduce measurement’s reliability. The infrapatellar fat pad is, in fact, a highly compliant and heterogeneous structure, characterized by variable composition (adipose tissue, fibrous septa, and vascular components), which can influence its mechanical response to compression. This intrinsic heterogeneity may contribute to variability in elastographic measurements. In addition, its marked deformability makes it particularly sensitive to even minimal transducer pressure, potentially introducing variability in strain distribution and increasing susceptibility to artifacts, especially in SE.

From a technical perspective, the anatomical location of the fat pad—superficial and adjacent to structures with different stiffness—may further affect wave propagation and signal stability, particularly in SWE, where boundary conditions and anisotropy can influence measurements.

Despite the large ROI that was chosen (the IPL diameter in transverse and the cranio-caudal thickness in longitudinal scans), both techniques showed good performances in repeatability and reproducibility.

The number of exams that were considered not suitable for evaluation was not different between the two techniques, so we can assume that feasibility of SE and SWE were similar. Nevertheless, since the ultrasound examination is known to be highly operator and experience dependent, it would be interesting to analyze feasibility of operators with different degrees of experience.

Despite the good performances in feasibility of the technique, SE performed better than SWE when a distinction between healthy and pathologic intermediate patellar ligaments was attempted. Indeed, a significant difference was found between GS and GL for the EI of the IPL, indicating that SE can aid discrimination. Nevertheless, Strain Ratio between IPL and the peripatellar fat was not statistically significant between GS and GL. This type of measure needs the use of an additional ROI to be drawn over a reference tissue, and this potentially introduces the opportunity of an error (37, 41).

As reported for SE at the distal insertion of the metacarpophalangeal joint capsule in horses (20), the EI was lower in diseased IPL in transverse scan and higher in longitudinal scan, meaning that the appearance of a diseased IPL is harder in transverse image and softer in longitudinal, probably as a result either of the pressure that was exerted by the operator or of the anisotropy of the ligament fibers.

Although SWE could not differentiate between GS and GL, we could observe that SWE variables (m/s and kPa) showed an inverse behavior compared to the distal insertion of the metacarpophalangeal joint capsule in horses (27), with higher velocity and rigidity in GL, suggesting harder tissue compared to that in GS. This result is similar to those from other studies where different anatomical regions (39) and techniques (42) were used, in both cases highlighting that tendon and ligament lesions modify their elastic properties according to their stage even in an experimental setting (42).

No correlation was found between any variable with the $\frac{\text{diameter}}{\text{thickness}}$ nor in GS or GL. This could indicate that the IPL does not react with hypertrophy to midbody lesions, or that a thinner IPL may be predisposed to injuries.

The small sample size and the numerical imbalance between the two groups represent two major limitations of the study. These constraints necessitated the use of non-parametric statistical analyses. Moreover, a more extensive analysis comparing the affected limb(s) of horses in the GL group with the unaffected contralateral limb(s) within the same group, or with limbs from the entire GS group, was considered unfeasible because of the marked imbalance between groups and the limited number of affected limbs.

A Doppler examination of the IPL was not performed. This could have improved interpretation of B-mode findings when a lesion was suspected (43–45) and possibly relate the degree of lameness to neovascularization (46). Although a microanatomical study on patellar ligament vascularization has recently been published (47), the current lack of knowledge about normal vascular architecture of normal intermediate patellar ligament does not allow reliable interpretation of a Doppler signal when present.

Patients’ selection is crucial to obtain consistent, reliable and repeatable results. Group allocation in our study relied on clinical presentation and B mode features of the IPL. As in previous studies, the horses belonging to GL had chronic moderate hindlimb lameness, without any history of trauma (7). The lack of a histologic description of the inspected ligament makes it difficult to assess how much the IPL contributed to the clinical manifestation of lameness, since it is known that its disorders usually occur in conjunction with other stifle injuries (3).

Despite the potential usefulness of SWE as an adjunctive diagnostic methodology in soft tissue pathologies (48, 49), this study showed that SE performed better. We could attribute these discrepancies to the anisotropic characteristics of tendons and ligament that limit optimal propagation of the acoustic impulse generated with SWE.

Although strain elastography (SE) and shear wave elastography (SWE) aim to assess similar tissue mechanical properties, they rely on different underlying principles. In particular, SWE is considered less operator-dependent but is more sensitive to tissue anisotropy, as shear wave propagation varies according to the orientation of collagen fibers relative to the ultrasound beam (50), potentially influencing measurement variability. In contrast, SE depends on externally applied compression and may introduce operator-related variability; however, in the present study this limitation was mitigated by the use of a real-time visual feedback system (traffic-light strain indicator), which provided an objective assessment of compression quality and contributed to standardizing data acquisition.

In conclusion, the equine IPL can be efficiently investigated with SE and SWE, as a supplementation to conventional diagnostic imaging techniques (i.e., radiography and B-Mode ultrasound). In both SE and SWE, the IPL has elastosonographic characteristic of a hard not deformable structure, with increased rigidity in chronic injuries. To support interpretation of abnormal grey scale images, the vasculature architecture in normal IPL should be investigated and described with Doppler ultrasound. Histology of investigated IPL could help to better characterize the elastosonographic appearance of this structure.

We have to acknowledge that the observed ability of strain elastography to differentiate healthy from diseased ligaments is based on a relatively small sample size and on a single parameter reaching statistical significance, which limits the strength and generalizability of our conclusions.

In light of this, these results should be considered preliminary and mainly hypothesis-generating. They suggest a potential role for strain elastography in this context, but they are not sufficient to support definitive clinical conclusions. In order to reduce the risk of type I error related to multiple comparisons, additional studies on larger and more heterogeneous cohorts are necessary to confirm the reproducibility and robustness of these results, as well as to clarify the diagnostic performance of strain elastography across different conditions. Longitudinal studies and comparisons with established reference standards would also be valuable to better define its clinical utility.

## Data Availability

The raw data supporting the conclusions of this article will be made available by the authors, without undue reservation.
